# Anticipated HIV Vaccine Acceptability among Sexually Active African-American Adult Women

**DOI:** 10.3390/vaccines1020088

**Published:** 2013-04-08

**Authors:** Julia Painter, Clare Cene-Kush, Alaina Conner, Carrie Cwiak, Lisa Haddad, Mark Mulligan, Ralph DiClemente

**Affiliations:** 1 Department of Behavioral Sciences and Health Education, Rollins School of Public Health, Emory University, 1518 Clifton Road, Atlanta, GA 30322, USA; 2 Vaccinology Training Program, School of Medicine, Emory University, 1462 Clifton Road, Atlanta, GA 30322, USA; 3 Department of Obstetrics and Gynecology, School of Medicine, Emory University, 1648 Pierce Drive NE, Atlanta, GA 30322, USA; 4 Division of Infectious Diseases, School of Medicine, Emory University, 1648 Pierce Drive NE, Atlanta, GA 30322, USA; 5 Department of Pediatrics, Emory University School of Medicine, 1648 Pierce Drive NE, Atlanta, GA 30322, USA

**Keywords:** HIV vaccine, women, attitudes, acceptability, behavioral theory

## Abstract

An HIV vaccine, once it becomes available, could reduce vulnerability to HIV among African-American women. The purpose of this study was to assess determinants of HIV vaccine acceptability among African-American women across hypothetical levels of vaccine efficacy. Participants were recruited from a hospital-based family planning clinic in Atlanta, GA serving low-income patients (N = 321). Data were collected from audio-computer assisted surveys administered in the clinic waiting room. Psychosocial survey items were guided by Risk Homeostasis Theory (RHT) and Social Cognitive Theory (SCT). Multivariate logistic regression was used to identify determinants of acceptability for two hypothetical HIV vaccines with 50% and 90% efficacy. Overall, 63% of participants would accept a vaccine with 50% efficacy and 85% would accept a vaccine with 90% efficacy. In multivariate analyses, odds of acceptability for a vaccine with 50% efficacy were higher among participants with greater perceived HIV vaccine benefits (AOR = 1.13, *p* < 0.001) and lower among participants with more than high school education (AOR = 0.47, *p* = 0.033) and greater perceived costs of HIV vaccination (AOR = 0.95, *p* = 0.010). Odds of acceptability for a vaccine with 90% efficacy were higher among participants with greater perceived costs of unprotected sex (AOR = 1.08, *p* = 0.026), HIV vaccine benefits (AOR = 1.23, *p* < 0.001) and self-efficacy for sex refusal (AOR = 1.11, *p* = 0.044). HIV vaccine acceptability was high, particularly for a vaccine with 90% efficacy. Findings suggest that demographic and psychosocial factors may impact acceptability of an eventual HIV vaccine. Once an HIV vaccine is available, interventions to maximize uptake may benefit from using RHT and SCT constructs to target relevant psychosocial factors, such as perceived benefits and perceived costs of vaccination.

## 1. Introduction

African-American women are disproportionately affected by HIV [1,2,3]. In 2009, an estimated 11,200 women were diagnosed with HIV in the U.S. [4], and the rate of new HIV infections among African-American women was 15 times the rate among White women [4,5]. African-American women are also disproportionately affected by STIs such as gonorrhea, chlamydia, trichomoniasis, and genital herpes (HSV-2), which can increase the risk of contracting HIV and transmitting HIV to others [6,7,8,9]. There is a clear need for effective interventions to reduce the burden of HIV among this population.

While several behavioral HIV-prevention interventions have demonstrated efficacy [10], these interventions alone may not be sufficient to control the spread of HIV. The introduction of a safe and effective HIV vaccine provides our best hope for ending the HIV pandemic [11], and would provide women, particularly African-American women, with much needed volitional control over vulnerability to HIV [12]. The promise of an effective vaccine has been supported by a recent vaccine trial in Thailand, which yielded a modest (30%), yet significant reduction in HIV infection, and several promising candidate vaccines are in the pipeline [13,14].

Yet an FDA licensed HIV vaccine may be futile if high-risk populations, such as African-American women, are not vaccinated. Little is known about whether African-American women would accept an HIV vaccine. A recent meta-analysis found 30 original studies (21 in the United States) that assessed HIV vaccine acceptability [15]. Among all populations assessed, (high-risk adults, MSM, prison inmates, adolescents, parents, college students, Asian/Pacific Islanders, and military personnel), the range of vaccine acceptability was 37% to 94%, with a mean acceptability of 65.3%. Factors associated with vaccine acceptability across studies included vaccine characteristics, structural factors, HIV vaccine attitudes, ethnicity, and risk group membership [15]. 

Of note, only three studies have focused exclusively on HIV vaccine acceptability among high-risk minority women [16,17,18], all of which were qualitative. Across studies, findings indicated that barriers to HIV vaccine acceptability among minority women include: concerns about vaccines in general, concerns about HIV vaccine safety and side effects (including reproductive side effects), mistrust of the medical system due to historical ethical controversies (such as the Tuskegee syphilis experiment), concerns about the perceptions of others/stigma, implications for sexual relationships, and affordability/insurance [16,17,18]. Motivators for getting an HIV vaccine included reduced worry about becoming infected with HIV, empowerment to protect oneself against HIV, and getting a recommendation from a health care provider [16,17,18].

Several additional studies, although not focused exclusively on women, have assessed HIV vaccine acceptability by gender [19,20,21,22,23]. Across studies, HIV acceptability concerns of particular salience to women included concerns about the HIV vaccine (such as past testing, safety, side effects), sex partner/relationship concerns, negative experiences with healthcare providers, affordability/insurance, and issues related to reproductive/teratogenic effects [19,20,21]. Gender-specific motivators included the ability to conceive a child without worrying about contracting HIV and support from sexual partners [19]. However, other studies did not identify gender-specific differences in HIV vaccine acceptability [22,23].

Although these studies provide valuable information, there is a need for larger, quantitative studies assessing anticipated HIV vaccine acceptability among African-American women. The aims of the present study were: (1) to assess anticipated HIV vaccine acceptability depending on varying HIV vaccine characteristics (such as efficacy and cost), and (2) to assess correlates of anticipated HIV vaccine acceptability under varying levels of vaccine efficacy (50% and 90%) among a sample of sexually active African-American women recruited from a hospital-based family planning clinic.

## 2. Experimental Section

### 2.1. Sample

Participants comprised African-American women presenting for clinical services at a hospital-based family planning clinic in Atlanta, GA, USA. To be eligible, participants had to self-identify as: African-American; women; aged 18*–*55 years; HIV-negative or unsure of HIV status; had at least one unprotected sex act (vaginal, anal, or oral) in the past 6 months; and provide verbal informed consent to participate in the study. This study was approved by the institutional review boards at the researchers’ institution and participating hospital.

### 2.2. Recruitment and Data Collection

Participants were recruited from the family planning clinic waiting room. Study staff approached women to ask if they were interested in participating in a research study, consisting of an hour-long survey with questions relating to an HIV vaccine. Interested patients were escorted to a private room for eligibility screening. Eligible participants completed audio-computer assisted (ACASI) surveys administered on laptop computers in a private room. The use of ACASI allowed participants to hear survey questions read through headphones, in addition to seeing them written on the computer screen. Study staff monitored survey administration and answered questions as needed. Most patients completed the survey prior to their clinic appointments. If participants were called for clinic appointments prior to survey completion, they were able to pause and resume their surveys after their appointments were finished. All participants who completed surveys received $25 cash as compensation.

### 2.3. Survey Instrument

The survey instrument was designed to investigate multiple factors potentially associated with HIV vaccine acceptability, including demographics, health history, sexual risk behaviors, personality traits, and theory-based psychosocial constructs.

HIV vaccine acceptability variables: HIV vaccine acceptability was assessed by asking the following questions: (1) How likely would you be to get an HIV vaccine that would prevent you from getting HIV about half the time (50% effective). (2) How likely would you be to get an HIV vaccine that would prevent you from getting HIV almost all the time (90% effective). Vaccine efficacies of 50% and 90% were chosen based on values used in previously published research [15,24,25]. (3) How likely would you be to get an HIV vaccine if it was available now. (4) How likely would you be to get an HIV vaccine if it required multiple doses (shots). (5) How likely would you be to get an HIV vaccine if it were free or covered by insurance. Participants initially answered questions on 5 point Likert scales ranging from 1 (Very unlikely) to 5 (Very likely). Answers were then dichotomized into “likely/very likely” compared to other answer choices. Additional questions assessed acceptability of out-of-pocket cost for the vaccine as well as who would influence participants’ decision to get the vaccine.

Background variables: Demographics included: age, education level, and receipt of public assistance in the past 12 months. Health history included: previous STD diagnosis, previous HIV test, and a brief mental health assessment (5 item Likert scale, higher scores indicate better mental health, alpha = 0.77) [26]. Measures of sexual risk behaviors included: Condom use at last sex, multiple vaginal sex partners in the past 3 months, and at least one casual partner for any sex (vaginal, anal, or oral) in the past 3 months. 

Personality variables: Personality variables included (1) sexual adventurism [27]; (2) sensation seeking and impulsivity [28]; and (3) positive future orientation [29]. All personality variables were measured on 5 point Likert scales, ranging from 1 (Strongly Disagree) to 5 (Strongly Agree). Scale details are presented in Table 1. 

**Table 1:** Personality and Psychosocial survey scale items.

Scale variables	# of items	Alpha	Items **
***Personality scale variables***			
Sexual adventurism	10	0.81	Having sex with a new partner is exciting to me
Sensation seeking and impulsivity	13	0.91	I like doing things just for the thrill of it
Positive future orientation	4	0.90	What happens to me in the future mostly depends on me
Psychosocial scale variables—RHT *			
Perceived benefits of unprotected sex	6	0.87	Having unprotected sex:
			Feels better than using a condom
			Is easier than using condom
			Makes me feel closer to my partner
			Shows my partner that I love him
			Shows my partner that I trust him
			Is pleasing to my partner
Perceived costs of unprotected sex	5	0.89	Having unprotected sex:
			Could cause me to get HIV
			Could cause me to get an STD
			Could put my partner at risk for HIV
			Could put my partner at risk for an STD
			Could make me feel guilty for putting my partner at risk for HIV
Perceived benefits of HIV vaccination—general	7	0.84	I would want an HIV vaccine because:
			The HIV vaccine could prevent me from getting HIV
			It would reduce my worry about getting HIV
			It would reduce my worry about giving HIV to my sex partner(s)
			It would reduce my worry about giving HIV to my children through childbirth or breastfeeding
			It would prevent me against getting HIV from unwanted sex or sexual assault
			I could tell my partners that I am protected against HIV
			My partner(s) would want me to get it
Perceived benefits of HIV vaccination—risk compensation	4	0.82	I would want an HIV vaccine because:
			It would reduce the hassle of using condoms
			It would allow me to have sex with a partner who is HIV positive
			It would allow me to take more sexual risks
			It would allow me to have sex with more sexual partners
Perceived costs of HIV vaccination—general	10	0.88	I would NOT want an HIV vaccine because:
			I believe the vaccine might give me HIV
			The vaccine may not work on me
			I do not like needles used by healthcare providers
			I would worry about vaccine safety or side effects
			It would cost too much money
			I am not at high risk for getting HIV
			I would still have to wear condoms to prevent getting other STDs
			I do not like any vaccines
			I would not want to get a newly developed vaccine
			I do not trust the medical community
Perceived costs of HIV vaccination—social norms	3	0.91	I would NOT want an HIV vaccine because:
			I would worry about what my friends think of me
			I would worry about what my family thinks of me
			I would worry about what my sex partner(s) thinks of me
Perceived HIV transmission risk	4	0.81	How often do you worry that you might get HIV?
Psychosocial scale variables—SCT *			
Peer norms supportive of unsafe sex	5	0.85	How many of your friends think that it’s okay to have sex without a condom?
Self-efficacy for sex refusal	7	0.86	How sure are you that you would be able to say NO to: Having sex with someone who refuses to wear a condom?
Barriers to condom negotiation	4	0.89	If I asked my partner to use a condom: He would think I don't trust him.

* RHT = Risk Homeostasis Theory; * SCT = Social Cognitive Theory; ** For newly developed measures, all scale items are provided. For existing validated scales, a sample item is given.

Psychosocial variables: Psychosocial variables were guided by two key theories: Risk Homeostasis Theory (RHT) [30,31] and Social Cognitive Theory (SCT) [32]. RHT, initially developed by Wilde, posits that humans have a subjective, target level of risk with which they are comfortable. This level of risk depends on (1) the expected benefits of the risky behavior, (2) the expected costs of the risky behavior, (3) the expected benefits of the safe behavior, and (4) the expected costs of the safe behavior [30,33]. People will compare their perceived level of risk with their target level of risk, and adjust their behavior to eliminate discrepancies. In 2007, Eaton and Kalichman proposed an adapted model of RHT to assess risk compensation associated with biomedical HIV prevention, including HIV vaccination. [31]. This study employed an RHT framework, as a person’s target level of risk may impact their decision to accept an HIV vaccine. SCT, developed by Bandura, takes into account the impact of environmental-level factors, (including interpersonal factors) that impact behavior. SCT was founded on the premise of reciprocal determinism, meaning that personal factors (ex. self-efficacy for sex refusal), environmental factors (ex. peer norms supportive of unsafe sex), and behavioral factors (ex. condom use) all influence each other, and thus can impact a person’s behavior. SCT is well-established, and has been shown to predict many heath behaviors, including vaccination [32,34]. This study also employed constructs from SCT to complement the RHT framework, and account for factors influencing HIV vaccine acceptability that may be unexplained by RHT alone. When possible, existing scales validated with African-American females were used. For previously untested constructs, additional scales were created by the authors and reviewed by content experts.

Psychosocial scale and index details are presented in Table 1. Psychosocial measures guided by RHT included: (1) perceived benefits of unprotected sex; (2) perceived costs of unprotected sex (3) perceived benefits of HIV vaccination in general; (4) perceived benefits of HIV vaccination in terms of risk compensation; (5) perceived costs of HIV vaccination in general; (6) perceived costs of HIV vaccination in terms of social norms; and (7) perceived HIV transmission risk [35]. All psychosocial variables guided by RHT were measured on 5 point Likert scales, ranging from 1 (Strongly Disagree) to 5 (Strongly Agree), except for perceived HIV transmission risk, which was measured on a 4 point Likert scale, ranging from 1 (Never) to 4 (Always). All RHT variables were scale variables, except for perceived costs of HIV vaccination in general, which was an index variable. Psychosocial measures guided by RHT were all developed for this study, except for HIV transmission risk. 

Psychosocial measures guided by SCT included: (1) peer norms supportive of unsafe sex, ranging from 1 (None) to 5 (All) [36]; (2) self-efficacy for sex refusal, ranging from 1 (Very hard to say no) to 5 (Very easy to say no) [36]; and (3) barriers to condom negotiation, ranging from 1 (Strongly Disagree) to 5 (Strongly Agree) [37].

### 2.4. Data Analysis

All analyses were conducted using SPSS version 19.0. Descriptive statistics were used to assess the distributions of all variables. Questions assessing psychosocial constructs were combined into scales, and Cronbach’s alphas were calculated to assess internal consistency. Bivariate analyses were used to assess associations between demographic, health history, behavioral, personality, and psychosocial factors with acceptability for an HIV vaccine with 50% efficacy and an HIV vaccine with 90% efficacy. Only variables that demonstrated significant bivariate associations at the *p* = 0.10 level were included in multivariate logistic regression analyses. For multivariate analysis, significance was measured at the *p* = 0.05 level.

## 3. Results

### 3.1. Response Rate

Of 623 women approached for participation, 508 (81.5%) were interested in screening, 353 (56.7%) were eligible to participate, and valid survey data were obtained from 321 (51.5%). The primary reason for ineligibility was not having unprotected sex (vaginal, anal, or oral) in the past 6 months, and the primary reason for declining to participate was not having enough time. The mean age of participants was 27.4 (SD = 7.7). In terms of education, 68 participants (21.2%) had not completed high school, 139 (43.3%) completed high school only, and 114 (35.5%) completed more than high school. The majority of the sample, (n = 252, 78.5%) received at least one type of public assistance in the past year.

### 3.2. Aim 1: Assessing HIV Vaccine Acceptability

More than half of all participants (n = 201, 62.6%) reported that they would accept a vaccine with 50% efficacy, and almost all (n = 274, 85.4%) would accept a vaccine with 90% efficacy. Among participants, 242 (75.3%) would get an HIV vaccine if it was available now, 222 (69.2%) would get an HIV vaccine if it required multiple doses (shots), and 259 (80.7%) would get an HIV vaccine if it were free or covered by insurance. Specifically, 112 participants (34.9%) would pay up to $50 out of pocket, 69 (21.5%) would pay up to $100 out of pocket, 65 (20.2%) would pay more than $100 out of pocket, 56 (17.4%) would only get it for free, and 19 (5.9%) would not get an HIV vaccine. People most likely to influence participants’ HIV vaccine decision-making were family members (n = 161, 50.2%), followed by doctors (n = 155, 48.3%), friends (n = 118, 36.8%), partners (n = 149, 16.4%), media (n = 28, 8.7%), and minister/preacher (n = 25, 7.8%). Further information regarding characteristics of participants willing to accept vaccines with varying levels of efficacy is displayed in Table 2.

**Table 2:** Characteristics of participants willing to accept vaccines with varying levels of efficacy.

Variables	Overall	Would not accept either vaccine	Willing to accept a vaccine with 50% efficacy	Willing to accept a vaccine with 90% efficacy
	(n = 321)	(n =46)	(n = 201)	(n = 274)
	n (%) or	n (%) or	n (%) or	n (%) or
	Mean (SD)	Mean (SD)	Mean (SD)	Mean (SD)
**Demographic variables**				
Age	27.4 (7.7)	28.5 (8.7)	27.6 (7.5)	27.3 (7.6)
Education				
Less than high school	68 (21.2)	10 (21.7)	48 (23.9)	57 (20.8)
High school	139 (43.4)	19 (41.3)	89 (44.3)	120 (43.8)
More than high school	114 (35.5)	17 (37.0)	64 (31.8)	97 (35.4)
Receipt of public assistance	252 (78.5)	33 (71.7)	162 (80.6)	218 (79.6)
**Health history variables**				
Previous STD diagnosis	210 (65.4)	22 (47.8)	134 (66.7)	187 (68.2)
Previous HIV test	306 (95.3)	45 (97.8)	194 (96.5)	260 (94.9)
Mental health	18.6 (3.7)	17.4 (3.5)	18.8 (3.7)	18.8 (3.7)
**Behavioral variables**				
Condom use at last sex	91 (28.3)	13 (28.3)	56 (27.9)	78 (28.5)
Multiple partners in the past 3 months	67 (20.9)	10 (21.7)	41 (20.4)	57 (20.8)
Casual partner in the past 3 months	95 (29.6)	15 (32.6)	62 (30.8)	80 (29.2)
**Personality variables**				
Sexual adventurism	23.9 (7.0)	24.7 (7.3)	23.9 (7.2)	23.8 (6.9)
Sensation seeking and impulsivity	28.8 (10.3)	28.1 (11.1)	28.6 (10.4)	28.9 (10.2)
Positive future orientation	17.3 (3.2)	16.2 (3.8)	17.4 (3.1)	17.5 (3.0)
Psychosocial variables—RHT *				
Perceived benefits of unprotected sex	17.6 (6.2)	15.9 (6.3)	17.7 (6.3)	17.9 (6.2)
Perceived costs of unprotected sex	21.0 (5.2)	18.1 (7.0)	21.4 (5.0)	21.5 (4.7)
Perceived benefits of HIV vaccination (general)	27.6 (5.9)	20.6 (6.6)	29.1 (4.7)	28.8 (4.9)
Perceived benefits of HIV vaccination (risk compensation)	8.5 (4.0)	8.9 (4.2)	8.6 (4.2)	8.4 (4.0)
Perceived costs of HIV vaccination (general)	22.9 (8.0)	26.2 (9.1)	21.7 (7.6)	22.5 (7.6)
Perceived costs of HIV vaccination (social norms)	5.2 (2.4)	6.2 (2.8)	4.9 (2.3)	5.0 (2.3)
HIV transmission worry	6.7 (2.7)	6.5 (2.3)	6.7 (2.7)	6.7 (2.8)
Psychosocial variables—SCT *				
Peer norms for unsafe sex	10.2 (4.4)	10.7 (4.7)	10.3 (4.6)	10.1 (4.4)
Self-efficacy for sex refusal	23.6 (4.2)	21.8 (4.7)	23.8 (4.1)	23.9 (4.0)
Barriers to condom negotiation	9.5 (4.5)	9.8 (4.7)	9.5 (4.6)	9.4 (4.4)

* RHT = Risk Homeostasis Theory; * SCT = Social Cognitive Theory.

### 3.3. Aim 2: Assessing Correlates of Anticipated HIV Vaccine Acceptability under Varying Levels of Vaccine Efficacy

Factors associated with acceptability of an HIV vaccine with 50% efficacy are presented in Table 3. In unadjusted bivariate analysis, the only demographic factor significantly associated with HIV vaccine acceptability at the *p* = 0.10 level was educational attainment. Participants with more than a high school education were less likely to accept an HIV vaccine with 50% efficacy compared to participants with less than a high school education. In terms of psychosocial variables, participants with greater perceived costs of unprotected sex, greater perceived benefits of HIV vaccination in general, and greater self-efficacy for sex refusal were more likely to accept an HIV vaccine with 50% efficacy. Participants with greater perceived costs of HIV vaccination in general and greater perceived social costs to HIV vaccination were less likely to accept an HIV vaccine with 50% efficacy. No health history, behavioral, or personality variables demonstrated significant associations.

**Table 3:** Factors associated with acceptability of an HIV vaccine with 50% efficacy.

Variables	Unadjusted Model	*p*-value	Adjusted Model	*p*-value
	OR (95%CI)		OR (95% CI)	
**Demographic variables**				
Age	1.01 (0.98, 1.04)	0.588	-	-
Education				
Less than high school	Reference	-		
High school	0.74 (0.40, 1.39)	0.350	0.59 (0.30, 1.18)	0.138
More than high school	0.53 (0.28, 1.01)	0.054	0.47 (0.23, 0.94)	0.033
Receipt of public assistance	1.39 (0.81, 2.38)	0.239	-	-
**Health history variables**				
Previous STD diagnosis	1.16 (0.72, 1.86)	0.544	-	-
Previous HIV test	1.98 (0.70, 5.60)	0.198	-	-
Mental health	1.03 (0.97, 1.10)	0.292	-	-
**Behavioral variables**				
Condom use at last sex	0.91 (0.55, 1.50)	0.716	-	-
Multiple partners in the past 3 months	0.90 (0.51, 1.57)	0.717	-	-
Casual partner in the past 3 months	1.17 (0.71, 1.94)	0.525	-	-
**Personality variables**				
Sexual adventurism	0.99 (0.96, 1.03)	0.702	-	-
Sensation seeking and impulsivity	1.00 (0.97, 1.02)	0.651	-	-
Positive future orientation	1.04 (0.97, 1.11)	0.325	-	-
Psychosocial variables—RHT *				
Perceived benefits of unprotected sex	1.01 (0.98, 1.05)	0.497	-	-
Perceived costs of unprotected sex	1.04 (1.00, 1.08)	0.069	1.00 (0.96, 1.06)	0.755
Perceived benefits of HIV vaccination (general)	1.14 (1.08, 1.19)	<0.001	1.13 (1.08, 1.20)	<0.001
Perceived benefits of HIV vaccination (risk compensation)	1.02 (096, 1.08)	0.508	-	-
Perceived costs of HIV vaccination (general)	0.94 (0.91, 0.97)	<0.001	0.95 (0.91, 0.99)	0.010
Perceived costs of HIV vaccination (social norms)	0.89 (0.81, 0.98)	0.019	1.08 (0.95, 1.23)	0.265
HIV transmission worry	1.00 (0.92, 1.09)	0.987	-	-
Psychosocial variables—SCT *				
Peer norms for unsafe sex	1.01 (0.95, 1.06)	0.841	-	-
Self-efficacy for sex refusal	1.05 (0.99, 1.11)	0.088	1.03 (0.97, 1.20)	0.320
Barriers to condom negotiation	1.02 (0.97, 1.07)	0.559	-	-

* RHT = Risk Homeostasis Theory; * SCT = Social Cognitive Theory.

In adjusted multivariate analysis, the odds of acceptability for an HIV vaccine with 50% efficacy were 1.13 times greater among participants who reported greater perceived benefits to HIV vaccination in general (*p* < 0.001). Women with more than a high school education and with higher perceived costs to HIV vaccination had a significant reduction in the odds of acceptability (*p* = 0.033 and *p* = 0.010, respectively). No other variables remained significant in multivariate analysis.

Factors associated with acceptability of an HIV vaccine with 90% efficacy are presented in Table 4. In unadjusted bivariate analysis, health history variables significantly associated with HIV vaccine acceptability at the *p* = 0.10 level included previous STD diagnosis and higher scores on the mental health scale. The only personality variable associated with acceptability of an HIV vaccine with 90% efficacy was positive future orientation. In terms of psychosocial variables, participants with greater perceived benefits of unprotected sex, greater perceived costs of unprotected sex, greater perceived benefits of HIV vaccination in general, and greater self-efficacy for sex refusal were more likely to accept an HIV vaccine with 90% efficacy. Participants with greater perceived costs of HIV vaccination in general and greater perceived social costs of HIV vaccination were less likely to accept an HIV vaccine with 90% efficacy. No demographic or behavioral variables demonstrated significant associations.

In adjusted multivariate analysis, the odds of acceptability for an HIV vaccine with 90% efficacy were 1.08 times larger among participants who reported greater perceived costs to unprotected sex (*p* = 0.026), 1.23 times larger among participants who reported greater perceived benefits to HIV vaccination in general (*p* < 0.001), and 1.11 times larger among participants with greater self-efficacy for sex refusal (*p* = 0.044). No other associations remained significant in multivariate analysis.

**Table 4:** Factors associated with acceptability of an HIV vaccine with 90% efficacy.

Variables	Unadjusted Model	*p*-value	Adjusted Model	*p*-value
	OR (95%CI)		OR (95% CI)	
**Demographic variables**				
Age	0.98 (0.95, 1.02)	0.375	-	-
Education				
Less than high school	Reference	-	-	-
High school	1.21 (0.54, 2.73)	0.631	-	-
More than high school	1.10 (0.48, 2.51)	0.819	-	-
Receipt of public assistance	1.48 (0.74, 3.01)	0.268	-	-
**Health history variables**				
Previous STD diagnosis	2.24 (1.20, 4.19)	0.011	1.71 (0.74, 3.68)	0.171
Previous HIV test	0.40 (0.05, 3.14)	0.386	-	-
Mental health	1.09 (1.01, 1.19)	0.021	1.08 (0.97, 1.20)	0.157
**Behavioral variables**				
Condom use at last sex	0.95 (0.47, 1.91)	0.895	-	-
Multiple partners in the past 3 months	0.89 (0.42, 1.92)	0.781	-	-
Casual partner in the past 3 months	0.88 (0.45, 1.71)	0.706	-	-
**Personality variables**				
Sexual adventurism	0.98 (0.94, 1.03)	0.458	-	-
Sensation seeking and impulsivity	1.01 (0.98, 1.04)	0.645	-	-
Positive future orientation	1.11 (1.02, 1.21)	0.015	1.00 (0.88, 1.15)	0.929
Psychosocial variables—RHT *				
Perceived benefits of unprotected sex	1.05 (1.00, 1.11)	0.045	1.05 (0.99, 1.13)	0.130
Perceived costs of unprotected sex	1.11 (1.05, 1.16)	<0.001	1.08 (1.01, 1.15)	0.026
Perceived benefits of HIV vaccination (general)	1.25 (1.17, 1.33)	<0.001	1.23 (1.14, 1.32)	<0.001
Perceived benefits of HIV vaccination (risk compensation)	0.97 (0.89, 1.04)	0.388	-	-
Perceived costs of HIV vaccination (general)	0.95 (0.91, 0.98)	0.006	0.96 (0.90, 1.02)	0.181
Perceived costs of HIV vaccination (social norms)	.085 (.075, 0.95)	0.006	1.07 (0.88, 1.31)	0.497
HIV transmission worry	1.03 (0.92, 1.16)	0.588	-	-
Psychosocial variables—SCT *				
Peer norms for unsafe sex	0.97 (0.90, 1.04)	0.427	-	-
Self-efficacy for sex refusal	1.12 (1.04, 1.20)	0.003	1.11 (1.00, 1.22)	0.044
Barriers to condom negotiation	0.99 (0.92, 1.06)	0.729	-	-

* RHT = Risk Homeostasis Theory; * SCT = Social Cognitive Theory.

## 4. Discussion

### 4.1. Discussion of Aim 1

The first aim of this study was to assess anticipated HIV vaccine acceptability depending on varying HIV vaccine characteristics. Previous studies have found that vaccine characteristics, such as efficacy, dose, and cost, are likely to impact HIV vaccine acceptability [38,39]. This study found that HIV vaccine acceptability was relatively high among participants across vaccine characteristics such as efficacy, dosage and cost. A majority of the women in this study would accept an HIV vaccine with 50% efficacy (n = 201, 63%), and a vast majority would accept an HIV vaccine with 90% efficacy (n = 274, 85%). While participants were more likely to accept a vaccine with 90% efficacy compared to 50% efficacy, many participants were likely to accept a vaccine with either scenario. This study also found that a majority of participants would accept an HIV vaccine even if it required multiple doses or out-of-pocket pay. In fact, more than 3 out of 4 participants were willing to pay at least $50 out of pocket, and 1 out of 5 participants were willing pay more than $100 out of pocket. These findings must be considered in the context of the population, the majority of which were unemployed and received some type of public assistance in the past year. There was clearly an interest and willingness to accept an HIV vaccine among the African-American women in this study. HIV vaccine acceptability was higher among the women in this study compared to previous studies conducted with minority women [16], although consistent with levels of acceptability from previous research across diverse populations [15].

These results are highly encouraging, particularly given the disproportionate burden of HIV and STIs among African-American women and the opportunity for volitional control over HIV risk that an HIV vaccine would present. Although anticipated vaccine acceptability does not necessarily portend vaccine uptake [40], results of this study indicate that African-American women may be likely to accept an FDA licensed vaccine against HIV.

### 4.2. Discussion of Aim 2

The second aim of this study was to assess correlates of anticipated HIV vaccine acceptability under varying levels of vaccine efficacy (50% and 90%). For an HIV vaccine with 50% efficacy, only one variable remained significantly associated with increased HIV vaccine acceptability in multivariate analyses: perceived benefits of HIV vaccination. This result is consistent with findings from previous qualitative studies indicating that perceived benefits of HIV vaccination, such as reduced worry about contracting HIV or transmitting HIV to other people, will be important motivators for vaccine uptake [16,17,18]. Two variables, higher educational attainment and high perceived costs of HIV vaccination, were associated with reduced HIV vaccine acceptability. It is understandable that participants with higher perceived costs to HIV vaccination (e.g., believing the vaccine might give them HIV, or not wanting a newly developed vaccine) would be less likely to accept an HIV vaccine. In terms of educational attainment, perhaps participants who were more educated were more likely to understand the implications of a vaccine with only 50% efficacy (e.g., they would still have to use condoms to prevent against HIV), and consequently less likely to accept the vaccine. Two of the significant correlates of HIV vaccine acceptability emerged from RHT, which underscores the salience of this model for understanding HIV vaccine acceptability.

For a vaccine with 90% efficacy, three variables were significantly associated with increased HIV vaccine acceptability in multivariate analysis: greater perceived costs of unprotected sex, perceived benefits of HIV vaccination, and greater self-efficacy for sex refusal. Two significant variables were psychosocial constructs from RHT (perceived costs of unprotected sex and perceived benefits of HIV vaccination), while one significant variable was a psychosocial construct from SCT (self-efficacy for sex refusal). This finding further supports the use RHT in the context of HIV vaccine acceptability, yet also highlights the potential importance of constructs from additional theories, such as SCT.

It is logical that women with greater perceived costs of unprotected sex, including putting themselves and their partners at risk for HIV, would be interested in getting a vaccine that would be highly efficacious. Although research among mixed-race adolescents has failed to find an association between perceived costs of unprotected sex and condom use [41,42], there is little known regarding the salience of this construct in increasing HIV protective behaviors among African-American adult women. Furthermore, getting an HIV vaccine is fundamentally different from condom use in multiple ways. First, getting an HIV vaccine would provide women with more volitional control over vulnerability to HIV compared to condom use. Second, depending on the dosing of the licensed vaccine, getting vaccinated against HIV would likely require a significantly shorter time investment than a potentially lifelong commitment to negotiating condom use. Third, getting vaccinated against HIV would not impact pleasure-related barriers to condom use, such as reduced feeling and ruining the mood. Thus, this construct may be of particular importance for increasing uptake of an HIV vaccine. 

Perceived benefits of HIV vaccination may also be a critical factor for HIV vaccine promotion interventions to address. It is important to note that perceived benefits of HIV vaccination was a significant predictor of HIV vaccine acceptability for vaccines with both 50% and 90% efficacy, even when controlling for other factors that were significant in bivariate analyses. This finding highlights the potential importance of scientific communication and marketing during an eventual HIV vaccine roll-out. Regardless of vaccine efficacy, a public health emphasis on increasing perceived benefits of vaccination may have a significant impact on vaccine uptake.

One additional construct, self-efficacy for sex refusal, was significantly associated with acceptability of an HIV vaccine with 90% efficacy. Previous research among African-American adolescent females has demonstrated an association between positive self-concept (including self-esteem, ethnic identity, and body image) and increased refusal of unprotected sexual intercourse [43]. Although the current study was conducted among African-American adult women, it is likely that positive self-concept continues to be associated with self-efficacy for sex refusal from adolescence into adulthood. It is also likely that women with more positive self-concept are likely to advocate for their sexual health in multiple ways, including refusing unwanted sex and getting vaccinated against HIV.

### 4.3. Limitations

First, this is a cross-sectional study. Thus, we could not assess the causal effects of the predictor variables on HIV vaccine acceptability. Second, the outcome is hypothetical HIV vaccine acceptability, not actual HIV vaccination. It may be difficult for women to predict how they will feel about a vaccine once it becomes available. Third, the data were self-reported, and possibly prone to social desirability bias. Fourth, the study population comprised sexually active African-American women in a low-income clinic setting in Atlanta, GA, USA. Thus, the results of this study may not be generalizable to broader populations. Furthermore, the study population was recruited from a clinical setting. African-American women receiving services from a hospital-based clinic may be more likely to accept biomedical HIV prevention interventions (such as vaccinations) compared to women who do not seek or receive health services. Fifth, although we can speculate reasons why women with higher educational attainment may be less likely to accept an HIV vaccine with 50% efficacy and why women with greater self-efficacy for sex refusal are more likely to accept a vaccine with 90% efficacy, we cannot fully explain these associations. Also, our index measure of perceived costs of HIV vaccination included ten distinct reasons why participants may not want an HIV vaccine. Future research is needed to determine which costs may be the most important barriers to HIV vaccination. Finally, for many of the variables that were significantly associated with HIV vaccine acceptability, the odds ratios were close to one. Thus, although the associations between these variables and HIV vaccine acceptability were statistically significant, it is unclear if these results would be clinically significant or practically meaningful in a public health context.

### 4.4. Implications

Findings from this study may be useful in identifying priority points to target during future HIV immunization campaigns. Although HIV vaccine acceptability was high overall, particularly for a vaccine with 90% efficacy, there was nonetheless variability regarding vaccine acceptability. Results of multivariate analyses for acceptability of HIV vaccines with 50% and 90% efficacy underscore the importance of vaccine-related perceptions, particularly perceived costs and benefits of vaccination, on HIV vaccine acceptability. Perceived costs of unprotected sex and self-efficacy for sex refusal may also be important determinants of acceptability for an HIV vaccine with 90% efficacy. Given that several psychosocial variables that emerged as significant for vaccines with both 50% and 90% efficacy were guided by RHT, our findings support the salience of this model for understanding HIV vaccine acceptability. Our findings also suggest it may be necessary to supplement the use of RHT with constructs from additional theories, such as SCT, to fully understand HIV vaccine acceptability. 

## 5. Conclusions

Once an HIV vaccine is available, intervention efforts to maximize uptake will need to address negative vaccine-related concerns and sufficiently communicate potential benefits. Interventions may also benefit from taking personal beliefs, such as perceived costs of unprotected sex and self-efficacy for sex refusal, into account. RHT and SCT-based constructs may be useful for guiding intervention efforts. Future studies should investigate factors associated with HIV vaccine acceptability associated with broader samples of African-American women, and women of all ethnicities.
